# Factors predicting organ-specific distant metastasis in patients with completely resected lung adenocarcinoma

**DOI:** 10.18632/oncotarget.11338

**Published:** 2016-08-17

**Authors:** Jung-Jyh Hung, Wen-Juei Jeng, Yu-Chung Wu, Teh-Ying Chou, Wen-Hu Hsu

**Affiliations:** ^1^ Division of Thoracic Surgery, Department of Surgery, Taipei Veterans General Hospital and School of Medicine, National Yang-Ming University, Taipei, Taiwan; ^2^ Department of Internal Medicine, Chang Gung Memorial Hospital and School of Medicine, Chang Gung University, Taipei, Taiwan; ^3^ Institute of Clinical Medicine, National Yang-Ming University, Taipei, Taiwan; ^4^ Department of Pathology and Laboratory Medicine, Taipei Veterans General Hospital, Taipei, Taiwan

**Keywords:** lung adenocarcinoma, survival, organ sites of metastasis, histology, subtype

## Abstract

The aim of the study is to demonstrate the relationship between clinicopathological variables and organ sites of metastasis in resected lung adenocarcinoma. The clinicopathological characteristics of 748 patients of resected lung adenocarcinoma at Taipei Veterans General Hospital between 2004 and 2012 were retrospectively reviewed. The prognostic value of clinicopathological variables for specific organ site metastasis-free survival was demonstrated. Among the 182 patients with distant metastasis, 93 (51.1%) patients developed contralateral lung metastasis, 81 (44.5%) had brain metastasis, 71 (39.0%) had bone metastasis, and 18 (8.9%) had liver metastasis during follow-up. Acinar predominant (Hazard ratio [HR], 0.468; 95% confidence interval [CI]: 0.250 to 0.877; *P* = 0.018) was significantly associated with less contralateral lung metastasis in multivariate analysis. Micropapillary predominant (HR, 2.686; 95% CI, 1.270 to 5.683; *P* = 0.010) was significantly associated with brain metastasis. Acinar predominant (HR, 0.461; 95% CI, 0.216 to 0.986; *P* = 0.046) was a significant prognostic factor for better contralateral lung metastasis-free survival in multivariate analysis. Micropapillary predominant (HR, 2.186; 95% CI, 1.148 to 4.163; *P* = 0.017) and solid predominant (HR, 4.093; 95% CI, 1.340 to 12.504; *P* = 0.013) were significant prognostic factors for worse brain metastasis-free survival and liver metastasis free-survival, respectively. There are significant differences in metastatic behavior between predominant pathological subtypes of lung adenocarcinoma. This information is important for patient follow-up strategy and identification of organ-specific distant metastasis. Prospective multi-institutional studies are mandatory for further validation.

## INTRODUCTION

Lung cancer is the main cause of cancer-related death worldwide [1]. Surgical resection is the treatment of choice for early-stage non-small cell lung cancer (NSCLC) [2]. Tumor recurrence after surgical resection is the most common cause of treatment failure [3–5]. Even with multimodality treatments, including chemotherapy, radiotherapy or a combination of other therapeutic modalities, most patients with recurrence after resection have little possibility of cure [3–5]. Identification of predictors for recurrence in patients with completely resected NSCLC is helpful for the use of adjuvant therapy or application of close follow-up strategy.

Organ tropism, also known as the seed-and-soil hypothesis, was first proposed by Stephen Paget in 1889 [6]. Solid tumors have great variation in patterns of metastatic organ tropism [7, 8]. A particular cancer will relapse in one particular organ or relapse in multiple specific organ sites [7, 8]. Many reports have demonstrated various predictors for organ-specific metastasis from solid tumors in the literature [9–11]. Association between breast cancer molecular subtypes and distinct pattern of metastasis has been reported [12–14]. The lung, brain and bone are the most common organ sites of metastasis in resected NSCLC [3–5, 15, 16]. In 2011, the International Association for the Study of Lung Cancer (IASLC), the American Thoracic Society (ATS), and the European Respiratory Society (ERS) proposed a new classification system of lung adenocarcinoma [17]. They recommended the use of comprehensive histological subtyping to assess histologic patterns semiquantitatively in 5% increments to choose a single predominant pattern (lepidic, acinar, papillary, micropapillary or solid) for invasive adenocarcinomas [17]. The significant prognostic value of the new classification on death and recurrence in lung adenocarcinoma has been reported and validated in many studies [18–21].

In our previous studies [22, 23], we have demonstrated that patients with micropapillary/solid predominant lung adenocarcinoma had significantly worse prognosis. We have also demonstrated that patients with micropapillary or solid predominant adenocarcinoma had a significantly higher possibility of developing initial extrathoracic-only recurrence than other subtypes [23]. However, whether the new classification predicts organ-specific metastasis of resected lung adenocarcinoma has not been investigated and reported in the literature. The study aims to demonstrate the predictors of organ-specific metastasis, including the new lung adenocarcinoma classification, in patients with completely resected lung adenocarcinoma with distant metastasis.

## RESULTS

The median follow-up time for all the 748 patients was 33.6 months (range, 3.2 to 113.1 months). The median number of mediastinal lymph node dissection/sampling was 18.0 (mean, 19.5 ± 9.2). The characteristics of these patients were listed in Table 1. For all patients, the 5-year overall survival and disease-free survival rates were 77.9% and 68.9%, respectively. Among the 182 patients with distant metastasis, there were 55 (30.2%) patients with acinar predominant, 43 (23.6.%) with papillary predominant, 44 (24.2%) with micropapillary predominant, and 40 (22.0%) with solid predominant adenocarcinoma. The median time to recurrence for the 182 patients was 15.2 months (range, 0.7 ± 93.4 months). Among the 182 patients, 93 (51.1%) patients developed contralateral lung metastasis, 81 (44.5%) had brain metastasis, 71 (39.0%) had bone metastasis, and 18 (8.9%) had liver metastasis during follow-up. Ninety-four (51.6%) of the 182 patients had local recurrence. For all 748 patients, the 2-year contralateral lung metastasis free-survival (CLMFS), brain metastasis free-survival (BrMFS), bone metastasis free-survival (BoMFS), and liver metastasis free-survival (LMFS) were 94.8%, 94.8%, 94.3%, and 98.5%, respectively.

**Table 1:** Clinicopathological variables of patients with resected lung adenocarcinoma

Variables	All Patients (*n* = 748)	Status of Distant Metastasis		
		No (*n* = 566)	Yes (*n* = 182)	*P* value
Age, years (mean ± SD)	63.2 ± 11.1	62.8 ± 11.3	64.2 ± 10.7	0.136
Sex, no. (%)				
Male	361 (48.3)	269 (47.5)	92 (50.5)	0.478
Female	387 (51.7)	297 (52.5)	90 (49.5)	
Tumor size, cm (mean ± SD)	2.6 ± 1.3	2.6 ± 1.1	3.5 ± 1.5	< 0.001
T status, no. (%)				
T1a	168 (22.5)	158 (27.9)	10 (5.5)	< 0.001
T1b	82 (11.0)	69 (12.2)	13 (7.2)	
T2a	427 (57.1)	307 (54.2)	120 (65.9)	
T2b	14 (1.8)	6 (1.1)	8 (4.4)	
T3	44 (5.9)	21 (3.7)	23 (12.6)	
T4	13 (1.7)	5 (0.9)	8 (4.4)	
N status, no. (%)				
N0	598 (79.9)	522 (92.2)	76 (41.8)	< 0.001
N1	59 (7.9)	22 (3.9)	37 (20.3)	
N2	91 (12.2)	22 (3.9)	69 (37.9)	
Stage, no. (%)				
IA	226 (30.3)	218 (38.5)	8 (4.4)	< 0.001
IB	330 (44.1)	277 (48.9)	53 (29.2)	
IIA	53 (7.1)	22 (3.9)	31 (17.0)	
IIB	29 (3.9)	18 (3.2)	11 (6.0)	
IIIA	107 (14.3)	31 (5.5)	76 (41.8)	
IIIB	3 (0.3)	0 (0.0)	3 (1.6)	
Visceral pleural invasion, no. (%)^*^				
Absent	293 (39.2)	249 (44.0)	44 (24.2)	< 0.001
Present	437 (58.4)	304 (53.7)	133 (73.1)	
Unknown	18 (2.4)	13 (2.3)	5 (2.7)	
Angiolymphatic invasion, no. (%)^*^				
Absent	517 (69.1)	440 (77.8)	77 (42.3)	< 0.001
Present	193 (25.8)	96 (17.0)	97 (53.3)	
Unknown	38 (5.1)	30 (5.2)	8 (4.4)	
Predominant pattern, no. (%)				
Lepidic predominant	74 (9.9)	74 (13.1)	0 (0.0)	< 0.001
Acinar predominant	302 (40.4)	247 (43.6)	55 (30.2)	
Papillary predominant	184 (24.6)	141 (24.9)	43 (23.6)	
Micropapillary predominant	105 (14.0)	61 (10.8)	44 (24.2)	
Solid predominant	83 (11.1)	43 (7.6)	40 (22.0)	
Adjuvant therapy, no. (%)				
No	461 (61.6)	392 (69.3)	69 (37.9)	< 0.001
Yes	287 (38.4)	174 (30.7)	113 (62.1)	

SD, Standard deviation.

* Patients with unknown status were excluded in the analysis.

### Association between organ sites of distant metastasis and clinicopathological variables

We first performed χ^2^ test and the paired independent sample *t*-test to investigate the relationship between specific organ sites of distant metastasis and clinicopathological variables (Table 2). Acinar subtype predominant (*P* = 0.005) was associated with less contralateral lung metastasis. Micropapillary (*P* = 0.002) and solid (*P* = 0.018) subtype predominant were associated with more contralateral lung metastasis. Micropapillary (*P* < 0.001) and solid (*P* = 0.024) subtype predominant were associated with brain metastasis. Micropapillary (*P* = 0.012) and solid (*P* < 0.001) subtype predominant were also associated with bone metastasis. Solid subtype predominant (*P* < 0.001) was associated with liver metastasis.

**Table 2:** Relationship between organ site of distant metastasis and clinicopathological variables in patients of resected lung adenocarcinoma

Variables	Contralateral Lung Metastasis			Brain Metastasis			Bone Metastasis			Liver Metastasis		
	No (*n* = 655)	Yes (*n* = 93)	*P* value	No (*n* = 667)	Yes (*n* = 81)	*P* value	No (*n* = 677)	Yes (*n* = 71)	*P* value	No (*n* = 730)	Yes (*n* = 18)	*P* value
Age, years (mean ± SD)	63.0 ± 11.2	64.5 ± 11.0	0.223	63.3 ± 11.2	61.7 ± 10.4	0.211	63.1 ± 10.6	63.6 ± 10.9	0.715	63.2 ± 11.1	62.1 ± 14.5	0.682
Sex, no. (%)												
Male	310 (47.3)	51 (54.8)	0.175	322 (48.3)	39 (48.1)	0.983	320 (47.3)	41 (57.7)	0.093	349 (97.8)	12 (66.7)	0.114
Female	345 (52.7)	42 (45.2)		345 (51.7)	42 (51.9)		357 (52.7)	30 (42.3)		381 (52.2)	6 (33.3)	
Tumor size, cm (mean ± SD)	2.4 ± 1.2	3.5 ± 1.6	< 0.001	2.5 ± 1.3	3.5 ± 1.5	< 0.001	2.4 ± 1.3	3.7 ± 1.4	< 0.001	2.5 ± 1.3	3.8 ± 1.5	< 0.001
N status, no. (%)												
N0 or N1	597 (91.1)	60 (64.5)	< 0.001	604 (90.6)	53 (65.4)	< 0.001	615 (90.8)	42 (59.2)	< 0.001	647 (88.6)	10 (55.6)	< 0.001
N2	58 (8.9)	33 (35.5)		63 (9.4)	28 (34.6)		62 (9.2)	29 (40.8)		83 (11.4)	8 (44.4)	
TNM stage, no. (%)												
I	522 (79.7)	34 (36.6)	< 0.001	528 (79.2)	28 (34.6)	< 0.001	530 (78.3)	26 (36.6)	< 0.001	550 (75.3)	6 (33.3)	< 0.001
II or III	133 (20.3)	59 (63.4)		139 (20.8)	53 (65.4)		147 (21.7)	45 (63.4)		180 (24.7)	12 (66.7)	
Visceral pleural invasion, no. (%)^*^												
Absent	273 (42.8)	20 (21.7)	< 0.001	273 (41.9)	20 (25.3)	0.004	277 (41.9)	16 (23.2)	0.003	289 (40.5)	4 (23.5)	0.158
Present	365 (57.2)	72 (78.3)		378 (58.1)	59 (74.7)		384 (58.1)	53 (76.8)		424 (59.5)	13 (76.5)	
Angiolymphatic invasion, no. (%)^*^												
Absent	474 (76.3)	43 (48.3)	< 0.001	483 (76.4)	34 (43.6)	< 0.001	491 (76.5)	26 (38.2)	< 0.001	512 (73.9)	5 (29.4)	< 0.001
Present	147 (23.7)	46 (51.7)		149 (23.6)	44 (56.4)		151 (23.5)	42 (61.8)		181 (26.1)	12 (70.6)	
Acinar predominant, no. (%)												
No	378 (57.7)	68 (73.1)	0.005	388 (58.2)	58 (71.6)	0.284	397 (58.6)	49 (69.0)	0.090	433 (59.3)	13 (72.2)	0.270
Yes	277 (42.3)	25 (26.9)		279 (41.8)	23 (28.4)		280 (41.4)	22 (31.0)		297 (40.7)	5 (27.8)	
Papillary predominant, no. (%)												
No	499 (76.2)	65 (69.9)	0.187	499 (74.8)	65 (80.2)	0.284	507 (74.9)	57 (80.3)	0.316	548 (75.1)	16 (88.9)	0.179
Yes	156 (23.8)	28 (30.1)		168 (25.2)	16 (19.8)		170 (25.1)	14 (19.7)		182 (24.9)	2 (11.1)	
Micropapillary predominant, no. (%)												
No	573 (87.5)	70 (75.3)	0.002	589 (88.3)	54 (66.7)	< 0.001	589 (87.0)	54 (76.1)	0.012	628 (86.0)	15 (83.3)	0.745
Yes	82 (12.5)	23 (24.7)		78 (11.7)	27 (33.3)		88 (13.0)	17 (23.9)		102 (14.0)	3 (16.7)	
Solid predominant, no. (%)												
No	589 (89.9)	76 (81.7)	0.018	599 (89.8)	66 (81.5)	0.024	612 (90.4)	53 (74.6)	< 0.001	655 (89.7)	10 (55.6)	< 0.001
Yes	66 (10.1)	17 (18.3)		68 (10.2)	15 (18.5)		65 (9.6)	18 (25.4)		75 (10.3)	8 (44.4)	

SD, Standard deviation.

* Patients with unknown status were excluded in the analysis.

### Logistic regression analysis for organ sites of distant metastasis

We further performed univariate and multivariate logistic regression analyses for specific organ sites of distant metastasis (Table 3). Greater tumor size (*P* = 0.006), N2 (vs. N0 or N1) (*P* = 0.046), and stage II or III (vs. stage I) (*P* = 0.002) were significantly associated with more contralateral lung metastasis in multivariate analysis. Acinar subtype predominant (Hazard ratio [HR], 0.468; 95% confidence interval [CI], 0.250 to 0.877; *P* = 0.018) was significantly associated with less contralateral lung metastasis. Greater tumor size (*P* = 0.017), stage II or III (vs. stage I) (*P* = 0.014), angiolymphatic invasion (*P* = 0.037), and micropapillary subtype predominant (HR, 2.686; 95% CI, 1.270 to 5.683; *P* = 0.010) were significantly associated with more brain metastasis in multivariate analysis. Greater tumor size (*P* = 0.002), N2 status (vs. N0 or N1) (*P* = 0.011), and angiolymphatic invasion (*P* = 0.003) were significantly associated with more bone metastasis in multivariate analysis. Angiolymphatic invasion (*P* = 0.031) was significantly associated with more liver metastasis in multivariate analysis. Solid subtype predominant (*P* = 0.059) showed a trend toward being significantly associated with more liver metastasis.

**Table 3:** Univariate and multivariate analyses of association between clinicopathological variables and organ sites of metastasis in patients of resected lung adenocarcinoma

Variables	Univariate			Multivariate		
	HR	95% CI	*P* value	HR	95% CI	*P* value
**Contralateral lung metastasis**						
Age^*^	1.012	0.993 to 1.032	0.223	1.007	0.984 to 1.031	0.557
Female	0.740	0.478 to 1.145	0.176	0.836	0.502 to 1.393	0.493
Tumor size^†^	1.681	1.446 to 1.953	< 0.001	1.306	1.081 to 1.579	0.006
N2 (vs. N0 or N1)	5.661	3.423 to 9.363	< 0.001	2.000	1.013 to 3.948	0.046
Stage II or III (vs. stage I)	6.811	4.287 to 10.821	< 0.001	3.034	1.519 to 6.059	0.002
Visceral pleural invasion	2.693	1.601 to 4.527	< 0.001	1.704	0.951 to 3.053	0.073
Angiolymphatic invasion	3.449	2.188 to 5.437	< 0.001	1.411	0.819 to 2.433	0.215
Acinar predominant	0.502	0.309 to 0.814	0.005	0.468	0.250 to 0.877	0.018
Papillary predominant	1.378	0.854 to 2.223	0.189			
Micropapillary predominant	2.296	1.358 to 3.881	0.002	0.868	0.425 to 1.775	0.699
Solid predominant	1.996	1.113 to 3.580	0.020	0.633	0.281 to 1.429	0.271
**Brain metastasis**						
Age^*^	0.987	0.967 to 1.007	0.211	0.985	0.961 to 1.010	0.230
Female	1.005	0.634 to 1.595	0.983	0.917	0.537 to 1.566	0.751
Tumor size^†^	1.593	1.367 to 1.856	< 0.001	1.276	1.045 to 1.559	0.017
N2 (vs. N0 or N1)	5.065	2.992 to 8.573	< 0.001	1.657	0.821 to 3.347	0.159
Stage II or III (vs. stage I)	7.190	4.385 to 11.790	< 0.001	2.469	1.201 to 5.076	0.014
Visceral pleural invasion	2.131	1.253 to 3.621	0.005	1.179	0.650 to 2.136	0.588
Angiolymphatic invasion	4.195	2.586 to 6.805	< 0.001	1.818	1.037 to 3.189	0.037
Acinar predominant	0.551	0.332 to 0.916	0.021	1.009	0.497 to 2.046	0.980
Papillary predominant	0.731	0.412 to 1.298	0.285			
Micropapillary predominant	3.776	2.247 to 6.343	< 0.001	2.686	1.270 to 5.683	0.010
Solid predominant	2.002	1.083 to 3.700	0.027	1.284	0.530 to 3.112	0.579
**Bone metastasis**						
Age^*^	1.004	0.982 to 1.026	0.715	0.995	0.969 to 1.022	0.726
Female	0.656	0.400 to 1.075	0.095	0.667	0.374 to 1.188	0.169
Tumor size^†^	1.762	1.496 to 2.075	< 0.001	1.388	1.127 to 1.710	0.002
N2 (vs. N0 or N1)	6.849	3.989 to 11.759	< 0.001	2.727	1.259 to 5.910	0.011
Stage II or III (vs. stage I)	6.240	3.724 to 10.457	< 0.001	1.481	0.653 to 3.356	0.347
Visceral pleural invasion	2.389	1.338 to 4.268	0.003	1.305	0.685 to 2.487	0.418
Angiolymphatic invasion	5.253	3.117 to 8.853	< 0.001	2.447	1.347 to 4.446	0.003
Acinar predominant	0.637	0.376 to 1.077	0.092	0.995	0.472 to 2.097	0.990
Papillary predominant	0.733	0.398 to 1.348	0.317			
Micropapillary predominant	2.107	1.169 to 3.799	0.013	1.459	0.627 to 3.391	0.380
Solid predominant	3.198	1.768 to 5.784	< 0.001	1.588	0.664 to 3.800	0.299
**Liver metastasis**						
Age^*^	0.991	0.951 to 1.034	0.682	0.985	0.939 to 1.033	0.527
Female	0.458	0.170 to 1.233	0.122	0.550	0.187 to 1.617	0.277
Tumor size^†^	1.586	1.234 to 2.038	< 0.001	1.255	0.879 to 1.791	0.212
N2 (vs. N0 or N1)	6.236	2.394 to 16.244	< 0.001	2.538	0.645 to 9.996	0.183
Stage II or III (vs. stage I)	6.111	2.261 to 16.518	< 0.001	1.163	0.235 to 5.757	0.853
Visceral pleural invasion	2.215	0.715 to 6.862	0.168			
Angiolymphatic invasion	6.789	2.359 to 19.536	< 0.001	3.599	1.126 to 11.507	0.031
Acinar predominant	0.561	0.198 to 1.589	0.276			
Papillary predominant	0.376	0.086 to 1.653	0.195			
Micropapillary predominant	1.231	0.350 to 4.329	0.746			
Solid predominant	6.987	2.675 to 18.245	< 0.001	2.929	0.958 to 8.954	0.059

HR, Hazard ratio; CI, confidence interval.

* The HR associated with age is that the increase in hazard is associated with a 1-year increase in age.

† The HR associated with tumor size is associated with a 1-cm increase in size.

### Analysis of specific organ sites metastasis-free survival

**Figure 1:**
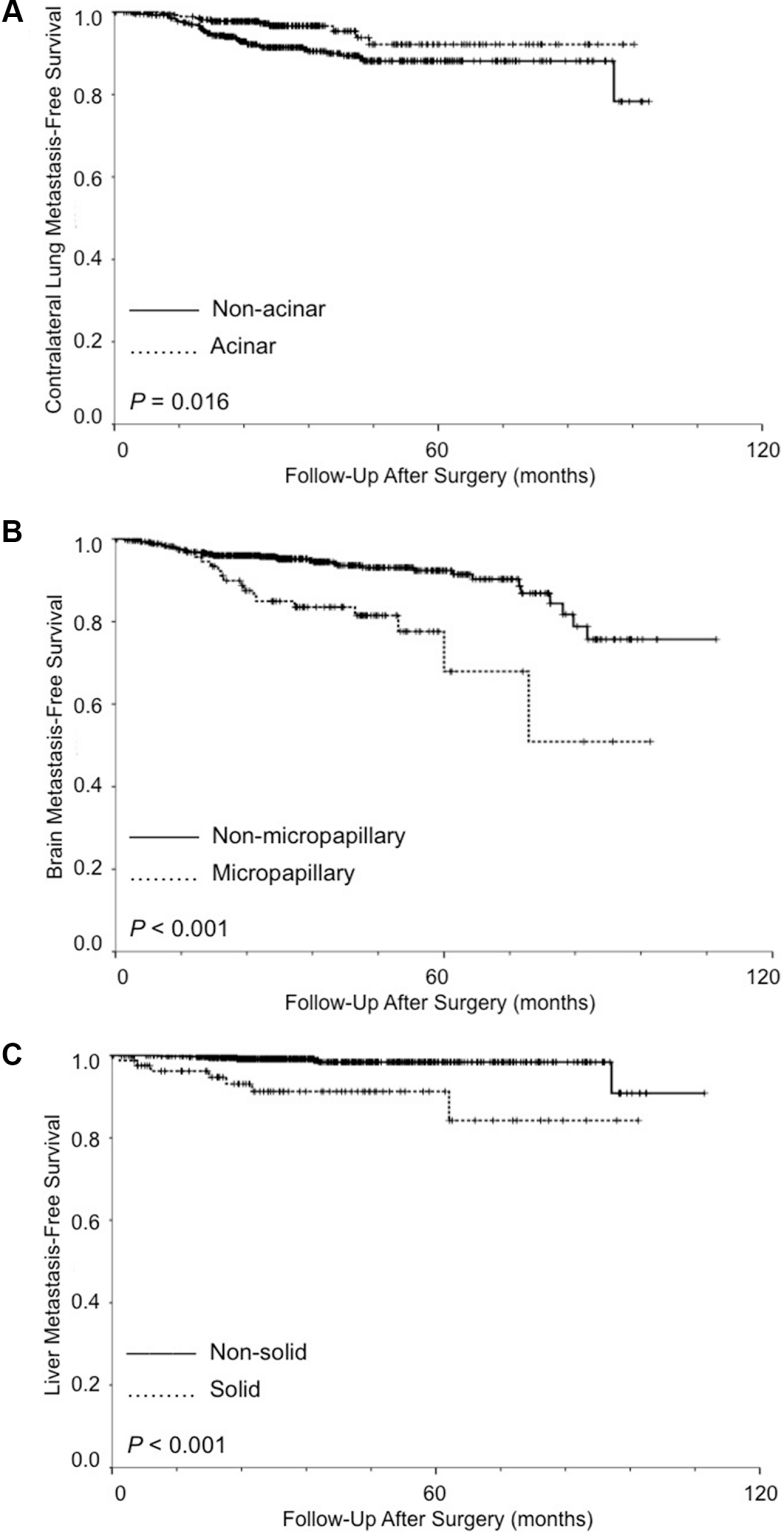
(**A**) Kaplan-Meier survival curves for contralateral lung metastasis-free survival stratified by acinar vs. non-acinar predominant adenocarcinoma. (**B**) Kaplan-Meier survival curves for brain metastasis-free survival stratified by micropapillary vs. non-micropapillary predominant adenocarcinoma. (**C**) Kaplan-Meier survival curves for liver metastasis-free survival stratified by solid vs. non-solid predominant adenocarcinoma. (Log-rank test).

In addition to univariate and multivariate logistic regression analyses, we performed univariate and multivariate analyses by Cox proportional hazards model to further demonstrate the prognostic factors for specific organ sites metastasis-free survival. Greater tumor size (HR, 1.293; 95% CI, 1.061 to 1.576; *P* = 0.011) was a significant prognostic factor for worse CLMFS, while acinar subtype predominant (HR, 0.461; 95% CI, 0.216 to 0.986; *P* = 0.046) was a significantly prognostic factor for better CLMFS in multivariate analysis (Table 4 and Figure 1A). For BrMFS, angiolymphatic invasion (HR, 2.632; 95% CI, 1.420 to 4.879; *P* = 0.002) and micropapillary subtype predominant (HR, 2.186; 95% CI, 1.148 to 4.163; *P* = 0.017) were significantly worse prognostic factors in multivariate analysis (Figure 1B). For BoMFS, female was a significantly better prognostic factor (HR, 0.571; 95% CI, 0.329 to 0.990; *P* = 0.046). Greater tumor size (HR, 1.268; 95% CI, 1.071 to 1.501; *P* = 0.006) and angiolymphatic invasion (HR, 2.993; 95% CI, 1.642 to 5.454; *P* < 0.001) were significantly worse prognostic factors in multivariate analysis. For LMFS, angiolymphatic invasion (HR, 3.699; 95% CI, 1.117 to 12.246; *P* = 0.032) and solid subtype predominant (HR, 4.093; 95% CI, 1.340 to 12.504; *P* = 0.013) was a significantly worse prognostic factor in multivariate analysis (Figure 1C).

**Table 4:** Univariate and multivariate analyses of specific organ sites metastasis-free survival in patients of resected lung adenocarcinoma

Variables	Univariate			Multivariate		
	HR	95% CI	*P* value	HR	95% CI	*P* value
**Contralateral lung metastasis-free survival**						
Age^*^	0.999	0.974 to 1.025	0.938	1.002	0.973 to 1.032	0.904
Female	1.314	0.743 to 2.324	0.348	1.337	0.728 to 2.455	0.349
Tumor size^†^	1.678	1.443 to 1.950	< 0.001	1.293	1.061 to 1.576	0.011
N2 (vs. N0 or N1)	5.110	2.828 to 9.234	< 0.001	1.373	0.634 to 2.972	0.421
Stage II or III (vs. stage I)	6.228	3.481 to 11.145	< 0.001	2.123	0.906 to 4.976	0.083
Visceral pleural invasion	2.053	1.068 to 3.948	0.031	0.991	0.475 to 2.067	0.981
Angiolymphatic invasion	4.089	2.313 to 7.228	< 0.001	1.796	0.924 to 3.490	0.084
Acinar predominant	0.446	0.228 to 0.875	0.019	0.461	0.216 to 0.986	0.046
Papillary predominant	1.161	0.624 to 2.160	0.637			
Micropapillary predominant	2.789	1.532 to 5.077	0.001	1.115	0.560 to 2.221	0.757
Solid predominant	1.725	0.808 to 3.684	0.159			
Adjuvant therapy	4.350	2.338 to 8.093	< 0.001	1.667	0.760 to 3.655	0.202
**Brain metastasis-free survival**						
Age^*^	0.993	0.970 to 1.015	0.520	0.996	0.971 to 1.022	0.783
Female	1.085	0.649 to 1.816	0.755	1.035	0.582 to 1.841	0.907
Tumor size^†^	1.498	1.304 to 1.721	< 0.001	1.176	0.974 to 1.420	0.092
N2 (vs. N0 or N1)	3.701	2.138 to 6.408	< 0.001	1.485	0.725 to 3.043	0.280
Stage II or III (vs. stage I)	4.823	2.865 to 8.121	< 0.001	1.795	0.803 to 4.009	0.154
Visceral pleural invasion	1.892	1.051 to 3.407	0.034	1.177	0.620 to 2.235	0.618
Angiolymphatic invasion	4.679	2.759 to 7.934	< 0.001	2.632	1.420 to 4.879	0.002
Acinar predominant	0.758	0.442 to 1.301	0.315			
Papillary predominant	0.617	0.320 to 1.189	0.149			
Micropapillary predominant	3.021	1.729 to 5.279	< 0.001	2.186	1.148 to 4.163	0.017
Solid predominant	2.032	1.029 to 4.013	0.041	1.939	0.863 to 4.356	0.109
Adjuvant therapy	2.540	1.511 to 4.270	< 0.001	0.698	0.345 to 1.410	0.316
**Bone metastasis-free survival**						
Age*	1.000	0.977 to 1.023	0.990	0.992	0.968 to 1.018	0.556
Female	0.598	0.355 to 1.007	0.053	0.571	0.329 to 0.990	0.046
Tumor size†	1.593	1.408 to 1.802	< 0.001	1.268	1.071 to 1.501	0.006
N2 (vs. N0 or N1)	4.694	2.774 to 7.942	< 0.001	1.497	0.754 to 2.973	0.249
Stage II or III (vs. stage I)	5.431	3.211 to 9.186	< 0.001	1.696	0.770 to 3.733	0.190
Visceral pleural invasion	2.644	1.404 to 4.977	0.003	1.497	0.758 to 2.954	0.245
Angiolymphatic invasion	5.791	3.397 to 9.874	< 0.001	2.993	1.642 to 5.454	< 0.001
Acinar predominant	0.661	0.380 to 1.149	0.143			
Papillary predominant	0.761	0.411 to 1.407	0.383			
Micropapillary predominant	2.048	1.139 to 3.683	0.017	1.324	0.683 to 2.567	0.406
Solid predominant	2.712	1.491 to 4.934	0.001	1.585	0.799 to 3.146	0.188
Adjuvant therapy	3.357	1.961 to 5.747	< 0.001	1.072	0.541 to 2.124	0.843
**Liver metastasis-free survival**						
Age^*^	0.994	0.950 to 1.040	0.803	0.983	0.935 to 1.035	0.518
Female	0.357	0.113 to 1.124	0.078	0.422	0.126 to 1.408	0.160
Tumor size^†^	1.601	1.234 to 2.077	< 0.001	1.242	0.846 to 1.823	0.268
N2 (vs. N0 or N1)	5.027	1.784 to 14.169	0.002	2.986	0.625 to 14.263	0.170
Stage II or III (vs. stage I)	4.130	1.458 to 11.697	0.008	0.576	0.093 to 3.551	0.552
Visceral pleural invasion	1.798	0.572 to 5.649	0.315			
Angiolymphatic invasion	5.794	1.975 to 16.996	0.001	3.699	1.117 to 12.246	0.032
Acinar predominant	0.577	0.183 to 1.817	0.348			
Papillary predominant	0.418	0.094 to 1.855	0.251			
Micropapillary predominant	0.943	0.211 to 4.212	0.939			
Solid predominant	7.390	2.676 to 20.407	< 0.001	4.093	1.340 to 12.504	0.013
Adjuvant therapy	3.289	1.122 to 9.647	0.030	1.556	0.412 to 5.872	0.514

HR, Hazard ratio; CI, confidence interval.

* The HR associated with age is that the increase in hazard is associated with a 1-year increase in age.

† The HR associated with tumor size is associated with a 1-cm increase in size.

### Application of the number of risk factors in predicting organ-specific metastasis in patients with stage I lung adenocarcinoma

**Figure 2:**
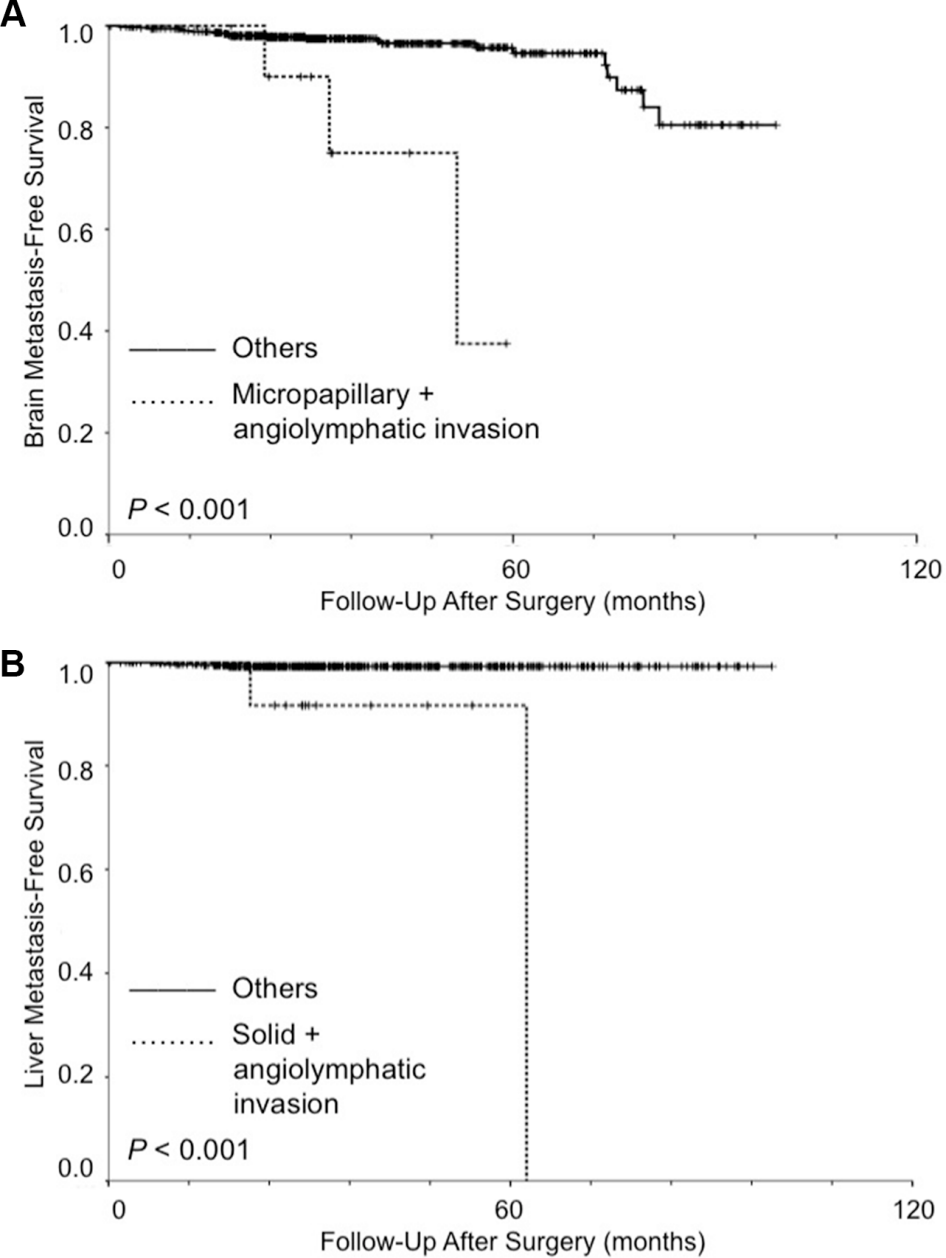
Kaplan-Meier analysis (log-rank test) for organ-specific metastasis in patients with stage I lung adenocarcinoma (**A**) For brain metastasis, the patients were divided into two groups according to number of positive risk predictors (micropapillary subtype predominant and angiolymphatic invasion): none positive or one positive (group 1) (*n* = 541), and two positive (group 2) (*n* = 15). Patients with both micropapillary subtype predominant and angiolymphatic invasion had significantly worse brain metastasis-free survival than others (*P* < 0.001). (**B**) For liver metastasis, the patients were divided into two groups according to number of positive risk predictors (solid subtype predominant and angiolymphatic invasion): none positive or one positive (group 1) (*n* = 540), and two positive (group 2) (*n* = 16). Patients with both solid subtype predominant and angiolymphatic invasion had significantly worse liver metastasis-free survival than others (*P* < 0.001).

To examine their cumulative predictive value on brain metastasis in stage I lung adenocarcinoma, micropapillary subtype predominant and angiolymphatic invasion were used as risk predictors for brain metastasis. All stage I patients (*n* = 556) were divided into two groups according to number of positive risk predictors: none positive or one positive (group 1) (*n* = 541), and two positive (group 2) (*n* = 15). Kaplan-Meier analysis showed that patients with both micropapillary subtype predominant and angiolymphatic invasion had significantly worse BrMFS than others (*P* < 0.001) (Figure 2A).

To examine their cumulative predictive value on liver metastasis in stage I lung adenocarcinoma, solid subtype predominant and angiolymphatic invasion were used as risk predictors for liver metastasis. All stage I patients (*n* = 556) were divided into two groups according to number of positive risk predictors: none positive or one positive (group 1) (*n* = 540), and two positive (group 2) (*n* = 16). Kaplan-Meier analysis showed that patients with both solid subtype predominant and angiolymphatic invasion had significantly worse LMFS than others (*P* < 0.001) (Figure 2B).

### Logistic regression analysis for organ sites of distant metastasis in the validation cohort

The median follow-up time for all the 438 patients in the validation cohort was 23.5 months (range, 0.1 to 37.8 months). For all these patients, the 3-year overall survival and disease-free survival rates were 96.0% and 94.0%, respectively. Twenty-two (5.0%) of the 438 patients developed distant metastasis during follow-up. Among the 22 patients with distant metastasis, there were 6 (27.3%) patients with acinar predominant, 2 (9.1%) with papillary predominant, 4 (18.2%) with micropapillary predominant, and 10 (45.5%) with solid predominant adenocarcinoma. The median time to recurrence for the 22 patients was 12.0 months (range, 3.3 ± 29.3 months). Among the 22 patients, 9 (40.9%) patients developed contralateral lung metastasis, 7 (31.8%) had brain metastasis, 8 (36.4%) had bone metastasis, and 3 (13.6%) had liver metastasis during follow-up. Eleven (50.0%) of the 22 patients had local recurrence.

We first performed univariate logistic regression analyses for specific organ sites of distant metastasis in the validation cohort (Supplementary Table 1). The backward stepwise regression procedure was used for multivariate analysis (Supplementary Table 2). N2 (vs. N0 or N1) (*P* < 0.001) was significantly associated with more contralateral lung metastasis in multivariate analysis. N2 (vs. N0 or N1) (*P* = 0.003) and micropapillary subtype predominant (HR, 8.035; 95% CI, 1.025 to 63.005; *P* = 0.047) were significantly associated with more brain metastasis. Stage II or III (vs. stage I) (*P* = 0.014) was significantly associated with more bone metastasis. Solid subtype predominant (*P* = 0.025) was significantly associated with more liver metastasis in univariate analysis. Multivariate analysis was not performed for liver metastasis due to the small number of patients with liver metastasis (*n* = 3).

## DISCUSSION

This study demonstrated that pathological subtypes of lung adenocarcinoma are associated with organ-specific metastasis in patients of resected lung adenocarcinoma with distant metastasis. Acinar, micropapillary, and solid predominant adenocarcinomas are significantly associated with contralateral lung metastasis, brain metastasis, and liver metastasis, respectively. Acinar, micropapillary and solid predominant adenocarcinomas are also significant prognostic factors for CLMFS, BrMFS and LMFS, respectively. By combination of risk factors, stage I patients with micropapillary subtype predominant and angiolymphatic invasion have significant worse BrMFS. Those with solid subtype predominant and angiolymphatic invasion have significant worse LMFS.

The new classification of lung adenocarcinoma proposed by IASLC/ATS/ERS in 2011 was a significant prognostic factor for survival and recurrence in lung adenocarcinoma [18–21]. In our previous study [23], we have demonstrated that patients with micropapillary or solid predominant adenocarcinoma had a significantly higher possibility of developing initial extrathoracic-only recurrence than other subtypes. While breast cancer molecular subtypes have been reported to predispose the site of distant metastases [12–14], whether the new classification of lung adenocarcinoma predicts organ-specific metastasis in completely resected lung adenocarcinoma remains unknown. In the current study, we showed that the new classification of lung adenocarcinoma is significantly associated with organ-specific metastasis in patients of resected lung adenocarcinoma with distant metastasis. Furthermore, we demonstrated the prognostic significance of the new classification in specific organ metastasis-free survival. Our study is the first to demonstrate the prognostic value of the new classification of lung adenocarcinoma for organ-specific metastasis in the literature.

The lung, brain and bone are the most common organ sites of metastasis in resected NSCLC [3–5, 15, 16]. In our previous study [5], bone was the most common site of single organ metastasis in patients with resected stage I NSCLC, followed by the brain. In the current study of resected stage I–III lung adenocarcinoma, contralateral lung was the most common site of metastasis, followed by the brain and the bone. We have demonstrated that acinar subtype predominant was associated with less contralateral lung metastasis, and was also a significant prognostic factor for better CLMFS. Approximately 10–25% of lung cancer patients have brain metastases at initial diagnosis [9]. About 40–50% of patients with lung cancer will develop brain metastases during the course of the disease [24, 25]. The brain is also one of the most common organ sites of metastasis in patients undergoing completely resected lung cancer [3–5, 12, 13]. Many biomarkers have been reported to be predictive for the development of brain metastases from lung cancer [9–11]. Several studies have demonstrated that brain metastases would be more frequent in patients with tumors harboring epidermal growth factor receptor (EGFR) mutations [26–28]. However, the numbers of patients were small in these studies, and were far too limited to make any firm conclusions. In our study, micropapillary predominant adenocarcinoma was significantly associated with more brain metastasis, and was a significant prognostic factor for worse BrMFS. For stage I lung adenocarcinoma, patients with both micropapillary subtype predominant and angiolymphatic invasion had significant worse BrMFS. Since our study is the first to demonstrate the association of the new classification of lung adenocarcinoma and organ-specific metastasis in the literature, we have conducted a validation cohort for confirmation. Although the number of patients with metastasis is small and the follow-up time was short in the validation cohort, micropapillary predominant adenocarcinoma was still significantly associated with more brain metastasis. The information is important and helpful to identify patients of resected lung adenocarcinoma who are at higher risk developing brain metastases because brain metastasis without neurologic symptoms may be difficult to be early diagnosed.

Although liver was not a common organ site of metastasis in lung cancer, liver metastasis was not rare during follow-up after surgery. Approximately 9% of patients with distant metastasis developed liver metastasis in our study. The predictor of liver metastasis from lung cancer has not been reported. In the study, we have demonstrated that solid predominant adenocarcinoma tended to be significantly associated with a higher frequency of liver metastasis. Solid predominant adenocarcinoma was a significant prognostic factor for worse LMFS. For stage I lung adenocarcinoma, patients with both solid subtype predominant and angiolymphatic invasion had significantly worse LMFS. In the validation cohort, solid subtype predominant adenocarcinoma was significantly associated with more liver metastasis in univariate analysis. Although multivariate analysis was not performed due to the small number of patients with liver metastasis, the association between solid predominant adenocarcinoma and liver metastasis was worthy of further investigation in the future. The results are important because patients with solid predominant adenocarcinoma may undergo more frequently liver imaging study during follow-up for early diagnosis of liver metastasis.

The associations between age, sex, tumor size, N status, tumor stage, visceral pleural invasion, angiolymphatic invasion, or adjuvant therapy and organ site of metastasis from lung cancer have not been well demonstrated. In the current study, female is a significant prognostic factor for better BoMFS. Greater tumor size was associated with more contralateral lung, brain, and bone metastasis. Greater tumor size was also a significant prognostic factor for worse CLMFS and BoMFS. N2 status (vs. N0 or N1) was associated with more contralateral lung and bone metastasis. Stage II or III (vs. stage I) was associated with more contralateral lung and brain metastasis. Angiolymphatic invasion was associated with more brain, bone, and liver metastasis. Angiolymphatic invasion was also a significant prognostic factor for worse BrMFS and BoMFS. No significant association between other conventional clinicopathological variables and organ sites of metastasis was identified.

There are some limitations and biases of this study that should be mentioned. As a retrospective single institute study, patient selection bias and time trend bias were inevitable. Another limitation is the diagnostic bias that conventional imaging would not necessarily detect all metastatic disease. Subclinical metastases may be missed simply because imaging not performed. There was also bias in defining a new primary lung cancer from a recurrent NSCLC. The major strength of this study is that a full and detailed follow-up of organ-specific metastasis made the analyses for specific organ site metastasis-free survival possible. Furthermore, we have conducted a validation cohort for internal validation. However, prospective multi-institutional studies are mandatory to further validate the prognostic value of the predominant pathological subtypes of lung adenocarcinoma on organ-specific metastasis after surgical resection.

In conclusion, this study demonstrates significant differences in metastatic behavior between predominant pathological subtypes of lung adenocarcinoma. Acinar predominant adenocarcinoma is associated with less contralateral lung metastasis. Micropapillary and solid predominant adenocarcinomas are significantly associated with brain and bone metastasis, respectively. This information is important for patient follow-up strategy and further study of molecular mechanisms leading to organ-specific metastasis in lung adenocarcinoma.

## MATERIALS AND METHODS

This study has been approved by the Institutional Review Board of Taipei Veterans General Hospital. From January 2004 to December 2012, all patients underwent completely resection for lung adenocarcinomas at Taipei Veterans General Hospital were retrospectively reviewed. Patients undergoing neoadjuvant chemotherapy or with stage IV disease were excluded. Patients with incomplete clinical information and follow-up were also excluded. A total of 801 patients were eligible for the study. Among them, 566 (70.7%) patients were free of tumor recurrence and 235 (29.3%) patients developed recurrence during follow-up. Only two patients with recurrence were diagnosed as lepidic predominant adenocarcinoma. The number of patients of lepidic predominant adenocarcinoma developing recurrence was small as compared with the other four subtypes of adenocarcinoma. Therefore, the 2 patients with lepidic predominant adenocarcinoma were excluded. Fifty-one (21.9%) of the remained 233 patients developed local only recurrence during follow-up, and was excluded for analysis. The remained 182 patients developed distant metastasis during follow-up. The 182 patients with distant metastasis and the 566 patients without tumor recurrence were included for analysis in the current study. The preoperative staging work-up was routinely performed as previously described [22, 23]. Mediastinoscopy was performed only when enlarged mediastinal lymph nodes (diameter > 1.0 cm) were shown by computed tomography scan. Complete resection of lung cancer and mediastinal lymph nodes dissection/sampling were performed as previously described [22, 23]. Determination of disease stages was based on the TNM classification (seventh edition) of the American Joint Committee on Cancer and the International Union Against Cancer [29, 30].

All resected specimens were formalin fixed and stained with hematoxylin and eosin, and were evaluated microscopically as previously described [23]. Each tumor was reviewed using comprehensive histological subtyping, recording the percentage of each histologic component (lepidic, acinar, papillary, micropapillary, and solid) in 5% increments as previously described [23]. The predominant pattern is defined according to the most dominant pattern.

All patients were followed-up at our outpatient department quarterly in the first 2 years after resection and semi-annually thereafter. The modalities and protocols during follow-up were used as previously described [5, 22, 23]. Computed tomography scans of chest and upper abdomen were routinely done in every scheduled outpatient department visit for follow-up. Nuclear medicine survey of the bone was arranged every 6 months in the first 2 years after resection and annually thereafter during follow-up. Suspicious bony lesions were confirmed by x-ray or bone biopsy. Computed tomography scan of brain was done when neurological symptoms occurred or when clinical suspicions were raised. Once a metastasis was discovered, a routine investigation was arranged to look for other metastatic sites. After initial diagnosis of recurrence, further examinations were arranged to discover other metastatic sites if symptoms occurred or clinical suspicions were raised. The hospital charts of all patients were reviewed to collect data of patterns of recurrence, organ sites of recurrence, and treatment for recurrence. Data collected from telephone call and correspondence letters during follow-up were also included.

To investigate their impact on specific organ site metastasis-free survival, clinicopathologic factors were examined in univariate and multivariate analyses. Local recurrence was defined as tumor recurrence in contiguous anatomical sites, including the ipsilateral hemithorax and mediastinum after surgical resection. Distant metastasis was defined as tumor recurrence in the contralateral lung or outside the hemithorax and mediastinum after surgical resection. Local only recurrence was defined as only local recurrence identified from initial operation to death or last follow-up. Distant only metastasis was defined as only distant metastasis discovered from initial operation to death or last follow-up. Secondary primary lung cancer was differentiated from recurrent NSCLC in patients undergoing surgical resection or biopsy according to the criteria proposed by Girard et al [31]. For those not undergoing resection or biopsy, judgment was made according to clinical course, eg. progression or aggressive clinical behavior (multiple lesions). The length of specific organ site (contralateral lung, brain, bone, or liver) metastasis-free survival was defined as the interval between the date of surgical resection and the date of the specific organ site metastasis (contralateral lung, brain, bone, or liver, respectively) or the last follow-up. An observation was censored at the last follow-up session when the patient was alive with specific organ metastasis-free status, or had died without specific organ metastasis.

To confirm the findings of the study, we conducted a validation cohort for further verification. From January 2013 to December 2014, all patients underwent completely resection for lung adenocarcinomas at Taipei Veterans General Hospital were retrospectively reviewed. The same exclusion criteria used in the original cohort were applied in the validation cohort. A total of 438 patients were eligible. Among them, 416 (95.0%) patients were free of tumor recurrence and 22 (5.0%) patients developed distant metastasis during follow-up.

The specific organ site metastasis-free survival was calculated by the Kaplan-Meier method [32]. The log-rank test was used to make group comparisons. To compare between groups with respect to categorical and continuous variables, the χ^2^ test and the paired independent sample *t*-test were used as appropriate. To investigate their association with specific organ sites of metastasis, clinicopathological factors were analyzed in univariate and multivariate logistic regression. For specific organ site metastasis-free survival, univariate and multivariate analyses were performed by means of the Cox proportional hazards model using SPSS software (version 20; IBM, Armonk, New York, USA). All variables with *P* < 0.1 in univariate analysis were entered into multivariate analysis. Age and sex were also entered for mutual adjustment despite *P* > 0.1. Statistical significance was defined as *P* < 0.05.

## SUPPLEMENTARY MATERIALS




