# Supplementary material for: Factors predicting organ-specific distant metastasis in patients with completely resected lung adenocarcinoma
Source: Oncotarget. 2016 Aug 17;7(36):58261–73. doi: 10.18632/oncotarget.11338 (PMC5295429; doi:10.18632/oncotarget.11338)
Supplement: Supplementary file 1 [file oncotarget-07-58261-s001.pdf]

Factors predicting organ-specific distant metastasis in patients with completely resected lung adenocarcinoma

Supplementary Materials

Supplementary Table S1: Univariate analysis of association between clinicopathological variables and organ sites of metastasis in the validation cohort of patients with resected lung adenocarcinoma. See Supplementary_Table_S1.

Supplementary Table S2: Multivariate analysis of association between clinicopathological variables and organ sites of metastasis in the validation cohort of patients with resected lung adenocarcinoma

Variables	Univariate		
	**HR**	**95% CI**	***P* value**
**Contralateral lung metastasis**			
N2 (vs. N0 or N1)	29.185	6.917 to 123.146	< 0.001
**Brain metastasis**			
N2 (vs. N0 or N1)	11.638	2.256 to 60.028	0.003
Micropapillary predominant	8.035	1.025 to 63.005	0.047
Solid predominant	5.598	0.811 to 38.631	0.081
**Bone metastasis**			
Stage II or III (vs. stage I)	5.918	1.442 to 24.294	0.014

HR, Hazard ratio; CI, confidence interval.
