# Supplementary material for: Factors predicting organ-specific distant metastasis in patients with completely resected lung adenocarcinoma
Source: Oncotarget. 2016 Aug 17;7(36):58261–73. doi: 10.18632/oncotarget.11338 (PMC5295429; doi:10.18632/oncotarget.11338)
Supplement: Supplementary file 2 [file oncotarget-07-58261-s002.docx]

Supplementary Table 1. Univariate analysis of association between clinicopathological variables and organ sites of metastasis in the validation cohort of patients with resected lung adenocarcinoma

|  | Univariate | | |
| --- | --- | --- | --- |
| Variables | HR | 95% CI | *P* value |
| **Contralateral lung metastasis** |  |  |  |
| Age* | 1.011 | 0.951 to 1.073 | 0.734 |
| Female | 0.231 | 0.048 to 1.127 | 0.070 |
| Tumor size† | 1.478 | 1.149 to 1.901 | 0.002 |
| N2 (vs. N0 or N1) | 29.704 | 7.040 to 125.326 | <0.001 |
| Stage II or III (vs. stage I) | 12.407 | 3.020 to 50.966 | <0.001 |
| Visceral pleural invasion | 1.464 | 0.647 to 3.315 | 0.360 |
| Angiolymphatic invasion | 6.825 | 1.676 to 27.794 | 0.007 |
| Acinar predominant | 0.679 | 0.167 to 2.749 | 0.587 |
| Papillary predominant | 6.524 | 1.264 to 44.659 | 0.025 |
| Micropapillary predominant | 3.216 | 0.644 to 16.075 | 0.155 |
| Solid predominant | 2.216 | 0.448 to 10.968 | 0.330 |
|  |  |  |  |
| **Brain metastasis** |  |  |  |
| Age* | 0.991 | 0.927 to 1.060 | 0.802 |
| Female | 0.620 | 0.137 to 2.802 | 0.534 |
| Tumor size† | 1.524 | 1.169 to 1.988 | 0.002 |
| N2 (vs. N0 or N1) | 18.437 | 3.938 to 86.316 | <0.001 |
| Stage II or III (vs. stage I) | 15.292 | 2.901 to 80.615 | 0.001 |
| Visceral pleural invasion | 1.735 | 0.693 to 4.344 | 0.240 |
| Angiolymphatic invasion | - | - | 0.993 |
| Acinar predominant | 0.224 | 0.027 to 1.874 | 0.167 |
| Papillary predominant | 3.614 | 0.414 to 31.538 | 0.245 |
| Micropapillary predominant | 4.526 | 0.847 to 24.182 | 0.077 |
| Solid predominant | 5.984 | 1.300 to 27.547 | 0.022 |
|  |  |  |  |
| **Bone metastasis** |  |  |  |
| Age * | 1.015 | 0.952 to 1.082 | 0.653 |
| Female | 0.830 | 0.205 to 3.361 | 0.794 |
| Tumor size† | 1.314 | 0.972 to 1.775 | 0.075 |
| N2 (vs. N0 or N1) | 4.280 | 0.829 to 22.095 | 0.083 |
| Stage II or III (vs. stage I) | 5.984 | 1.458 to 24.562 | 0.013 |
| Visceral pleural invasion | 2.580 | 1.055 to 6.305 | 0.038 |
| Angiolymphatic invasion | 3.333 | 0.819 to 13.571 | 0.093 |
| Acinar predominant | 1.376 | 0.340 to 5.573 | 0.655 |
| Papillary predominant | 3.090 | 0.362 to 26.401 | 0.303 |
| Micropapillary predominant | 3.762 | 0.732 to 19.339 | 0.113 |
| Solid predominant | 1.086 | 0.131 to 9.009 | 0.939 |
|  |  |  |  |
| **Liver metastasis** |  |  |  |
| Age* | 0.930 | 0.853 to 1.015 | 0.104 |
| Female | 0.414 | 0.037 to 4.598 | 0.473 |
| Tumor size† | 1.243 | 0.738 to 2.094 | 0.413 |
| N2 (vs. N0 or N1) | 26.000 | 2.293 to 294.768 | 0.009 |
| Stage II or III (vs. stage I) | 11.683 | 1.044 to 130.762 | 0.046 |
| Visceral pleural invasion | 6.045 | 0.868 to 42.107 | 0.069 |
| Angiolymphatic invasion | - | - | 0.994 |
| Acinar predominant | - | - | 0.995 |
| Papillary predominant | 10.947 | 0.950 to 126.115 | 0.055 |
| Micropapillary predominant | - | - | 0.998 |
| Solid predominant | 15.755 | 1.403 to 176.965 | 0.025 |

HR, Hazard ratio; CI, confidence interval. *The HR associated with age is that the increase in hazard is associated with a 1-year increase in age. †The HR associated with tumor size is associated with a 1-cm increase in size.
